# Phylogenomic analysis of *Mycoplasma bovis* from Belgian veal, dairy and beef herds

**DOI:** 10.1186/s13567-020-00848-z

**Published:** 2020-09-23

**Authors:** Jade Bokma, Nick Vereecke, Koen De Bleecker, Jozefien Callens, Stefaan Ribbens, Hans Nauwynck, Freddy Haesebrouck, Sebastiaan Theuns, Filip Boyen, Bart Pardon

**Affiliations:** 1 Department of Large Animal Internal Medicine, Faculty of Veterinary Medicine, Ghent University, Salisburylaan 133, 9820 Merelbeke, Belgium; 2 Department of Virology, Parasitology and Immunology, Faculty of Veterinary Medicine, Ghent University, Salisburylaan 133, 9820 Merelbeke, Belgium; 3 DGZ (Animal Health Service-Flanders), Industrielaan 29, 8820 Torhout, Belgium; 4 Department of Pathology, Bacteriology and Avian Diseases, Faculty of Veterinary Medicine, Ghent University, Salisburylaan 133, 9820 Merelbeke, Belgium

**Keywords:** cattle, long-read nanopore sequencing, phylogenetic analysis, SNP analysis, whole genome

## Abstract

*M. bovis* is one of the leading causes of respiratory disease and antimicrobial use in cattle. The pathogen is widespread in different cattle industries worldwide, but highest prevalence is found in the veal industry. Knowledge on *M. bovis* strain distribution over the dairy, beef and veal industries is crucial for the design of effective control and prevention programs, but currently undocumented. Therefore, the present study evaluated the molecular epidemiology and genetic relatedness of *M. bovis* isolates obtained from Belgian beef, dairy and veal farms, and how these relate to *M. bovis* strains obtained worldwide. Full genomes of one hundred Belgian *M. bovis* isolates collected over a 5-year period (2014–2019), obtained from 27 dairy, 38 beef and 29 veal farms, were sequenced by long-read nanopore sequencing. Consensus sequences were used to generate a phylogenetic tree in order to associate genetic clusters with cattle sector, geographical area and year of isolation. The phylogenetic analysis of the Belgian *M. bovis* isolates resulted in 5 major clusters and 1 outlier. No sector-specific *M. bovis* clustering was identified. On a world scale, Belgian isolates clustered with Israeli, European and American strains. Different *M. bovis* clusters circulated for at least 1.5 consecutive years throughout the country, affecting all observed industries. Therefore, the high prevalence in the veal industry is more likely the consequence of frequent purchase from the dairy and beef industry, than that a reservoir of veal specific strains on farm would exist. These results emphasize the importance of biosecurity in *M. bovis* control and prevention.

## Introduction

*Mycoplasma bovis* (*M. bovis*) causes mostly pneumonia, arthritis, otitis in calves and mastitis in adult cattle [1, 2] resulting in high antimicrobial use (AMU) and enormous economic losses in cattle farming sectors worldwide [2–4]. In Belgium, 100% of the veal farms are seropositive for *M. bovis* [5, 6], whereas *M. bovis* is involved in 33% of acute pneumonia outbreaks in beef and dairy farms [7]. Treatment of *M. bovis* is frequently unsatisfactory, probably due to a combination of intrinsic and acquired antimicrobial resistance, immuno-evasive properties of the pathogen and failure of the animal to generate an effective immune response [8, 9]. Together with the absence of an effective commercially available vaccine, the control of *M. bovis* is particularly challenging.

A contemporary fear is that the veal sector, currently combining a high AMU and a farm level *M. bovis* prevalence of 100%, is a reservoir for multi-resistant sector-specific *M. bovis* strains [6, 10, 11]. Currently, there is insufficient knowledge about the epidemiology of circulating *M. bovis* strains to answer this question. Several epidemiological studies observed clonal emergence and identified dominant lineages of the *M. bovis* bacterium, based on antimicrobial resistance patterns and different strain typing methods [12–15]. In the past, different approaches were used to subtype *M. bovis* strains, including random amplification of polymorphic DNA (RAPD), arbitrarily primed PCR (AP-PCR), amplification fragment length polymorphism (AFLP), pulsed-field gel electrophoresis (PFGE), multiple-locus variable-number tandem repeat (MLVA), and multi-locus sequence typing (MLST) [13, 14, 16–18]. Unfortunately, results from these typing methods are difficult to compare and only focus on a small fraction of the genomic information, resulting in a limited insight in the genetics and incongruence among studies [17, 19, 20]. Therefore, whole genome sequencing (WGS) could be a great opportunity, considering its highly discriminative capacity and reproducibility compared to older typing methods [21, 22].

Several studies already investigated whether specific *M. bovis* strains were associated with affected organs, such as udder, respiratory tract or joints [14, 23, 24], geographical location [23, 24] or health status [23, 25]. Only one study determined epidemiology based on AP-PCR in three farms from three different husbandry conditions (dairy calf ranch, feedlot and closed beef herd), presuming that management factors could influence the distribution of *M. bovis* [16]. These husbandry conditions are not comparable with the three main sectors in Europe. In Europe, a lot of short-distance movements of cattle between farms is seen, and the veal industry is an important side market of the dairy and beef industry [26, 27]. It is currently not clear whether sector-specific *M. bovis* strains are present and what their genetic relation is to previously sequenced *M. bovis* isolates. Therefore, the present study first evaluated the molecular epidemiology and genetic relatedness of *M. bovis* isolates obtained from Belgian beef, dairy and veal farms. Furthermore, it studied the relationship of these isolates to *M. bovis* strains from other countries.

## Materials and methods

### *Mycoplasma bovis* collection and identification

One hundred *M. bovis* isolates were obtained from 94 Belgian farms (27 dairy, 38 beef and 29 veal) over a 5-year period (2014–2019). All isolates were obtained from diagnostic samples collected by field veterinarians from clinical cases, in compliance with EU legislation on ethics in animal experimentation [2010/63/EU]. Isolates were collected in 2014 (n = 1), 2016 (11), 2017 (63), 2018 (19) and 2019 (6), originating from the provinces East-Flanders (n = 10), West-Flanders (25), Antwerp (38), Limburg (6), Flemish Brabant (10), Heynowes (6), Namen (2) and Liege (1). The origin of two isolates was unclear. The samples were retrieved from the respiratory tract (89), middle ear (3), milk (4), joint (2) and other fluids (3) of calves and adult cattle, as shown in Additional file 1. The samples were cultured on selective indicative agar [28], and identification was verified with MALDI-TOF MS (score value ≥ 1.7), as described earlier [29] and Kraken2 analysis. Isolates had a passage history of maximum 3–5 times and all isolates were stored at maximum − 20 °C until further analysis.

### Preparation and DNA extraction

In ten separate runs, all *M. bovis* isolates were thawed and cultured in 10 mL modified PPLO broth (pH 7.8) (Difco™, BD Diagnostic Systems, Sparks, Md.), supplemented with 25% inactivated horse serum (Gibco™), 0.7% technical yeast extract, 0.5% sodium pyruvate (ReagentPlus, Sigma-Aldrich®), 0.5% D-( +)-glucose monohydrate (Sigma-Aldrich) and 0.005% phenol red. After 4 days of incubation (37ºC, 5% CO_2_) a bacterial suspension of approximately 10^8^ CFU/mL was obtained. Bacterial DNA was obtained using the ZymoBIOMICS DNA Miniprep kit (Zymo Research) according to the manufacturer’s instructions. Quantity and quality were verified using NanoDrop ND-1000 spectrophotometer (Thermo Scientific). Low quality samples were further cleaned using CleanNGS (CleanNa) beads. All runs included the *M. bovis* PG45 type strain (ATCC 25523) and modified PPLO broth as the positive and negative control, respectively.

### Library preparation and MinION long-read sequencing

Quality-checked native *M. bovis* DNA was immediately used for library preparation using the Rapid Barcoding Sequencing Kit (SQK-RBK004; Oxford Nanopore Technologies (ONT)), following manufacturers’ instructions. For each run, ten field strains, one positive control (PG45) and one negative control (sterile broth) were multiplexed (400 ng DNA per sample). A new R9.4.1 Flow cell (ONT) was used for a 48 h sequencing run on MinION device (ONT). Raw fast5 read files were collected using MinKnow v.3.6.5.

### Bioinformatics pipeline

All data were analyzed on an Ubuntu 18.04.3 LTS system. In order to speed up bioinformatics analyses, GPU resources (GeForce RTX 2080 Ti/PCIe/SSE2) were exploited where possible. Raw fast5 files were basecalled using Guppy basecaller (GPU v.3.3.0; ONT), followed by demultiplexing, adapter trimming, and quality filtering (Q-score ≥ 7) of fastq files with qcat (v.1.1.0; ONT) and NanoFilt (v. 2.5.0; [30]), respectively. Reference-based assemblies were generated using the *M. bovis* PG45 type strain sequence (NC_014760.1) by mapping filtered reads onto the reference using GraphMap (v.0.5.2; [31]). Final consensus sequences were generated using Medaka (GPU v.0.10.0; ONT). All strains were identified as *M. bovis* using Kraken2 (v2.0.8; [32]) by aligning the reads against the minikraken_v1_8GB database with standard settings. Overall consensus assembly accuracies were verified by comparing total Single Nucleotide Polymorphisms (SNPs) using the CSI phylogeny package (v1.4, Center for Genomic Epidemiology, Denmark; [33]) as compared to the *M. bovis* PG45 type strain (NC_014760.1) reference sequences. To validate the use of long-read sequencing, SNPs of ten independent *M. bovis* PG45 assemblies were compared to those in a single MiSeq experimental dataset. All *M. bovis* consensus genomes are available for download on the NCBI GenBank database under the BioProject PRJNA639688 and accession numbers (SAMN15246515-SAMN1524662). Sequencing summaries can be found in Additional file 1.

### Phylogenetic analysis

Phylogenetic analysis was performed on all newly generated consensus sequences alone or in combination with 250 previously published *M. bovis* sequences using the FastTree-based CSI Phylogeny v1.4 (see Additional file 2). All analyses included the *M. bovis* PG45 type strain (NC_014760.1) as reference and *M. agalactiae* PG2 (NC_009497.1) as outgroup. Resulting Newick files were visualized with MEGA-X software [34].

### Cluster and strain determination

Due to the lack of relatedness criteria for SNP typing schemes of *M. bovis* and the need to establish these per organism and experimental design [35], clusters were defined by visual inspection of the phylogenetic tree and by taking into account bootstrap support. In addition, the matrix of pairwise SNP counts was extracted from CSI Phylogeny for further inspection. Mean SNP differences were calculated between within-cluster isolates, and outliers were defined with the 1.5xIQR rule, using the Outlier Calculator (https://miniwebtool.com/outlier-calculator/).

### Geographical distribution

Esri®ArcMap™ (version 10.7.1) software was used to visualize the geographical distribution and density of *M. bovis* isolates over Belgium. Herd size was based on the national Identification and Registration system, containing, on the first of January 2017, a total of 23 995 cattle herds in Belgium (23 733 conventional herds; 262 veal), and a total of 2 517 850 cattle. The spatial distribution of the Belgian cattle (both cattle and veal calves) was displayed using kernel smoothing. Coordinates of Belgian cattle herds were converted into a continuous raster using the kernel density estimation function weighted by number of cattle (Spatial Analyst, ArcMAP X, ESRI, Redlands, CA, USA).

## Results

### Phylogenetic analysis of Belgian isolates

A median sequencing depth of 618X (range: 32X-2689X) was obtained from long reads with an average N_50_ read length of 5706 ± 1514 bp for all Belgian *M. bovis* isolates. First, implementation of long-read sequencing of *M. bovis* genomes was validated by comparing total SNPs from ten independently sequenced *M. bovis* PG45 sequences and a single *M. bovis* PG45 MiSeq dataset, showing 53 ± 3 SNPs and 27 SNPs difference, respectively, compared with the 1 003 404 bp of the *M. bovis* PG45 reference genome (NC_014760.1). The observed average SNP difference of 0.005% for the long-read sequencing approach was considered acceptable to allow meaningful phylogenetic analyses. In addition, control strain *M. bovis* PG45 results were mutually compared over all ten runs, showing a mean SNP difference of 20 (range 8–30, standard deviation 4.6), which was also demonstrated an acceptable inter-experimental variation.

Taking into account all Belgian *M. bovis* strains, and also including the outgroup *M. agalactiae,* 51.4% of the *M. bovis* genome or 515 324 nucleotide positions were used for phylogeny. The minimum and maximum SNP differences among Belgian *M. bovis* isolates were 33 and 3775, respectively.

**Figure 1:**
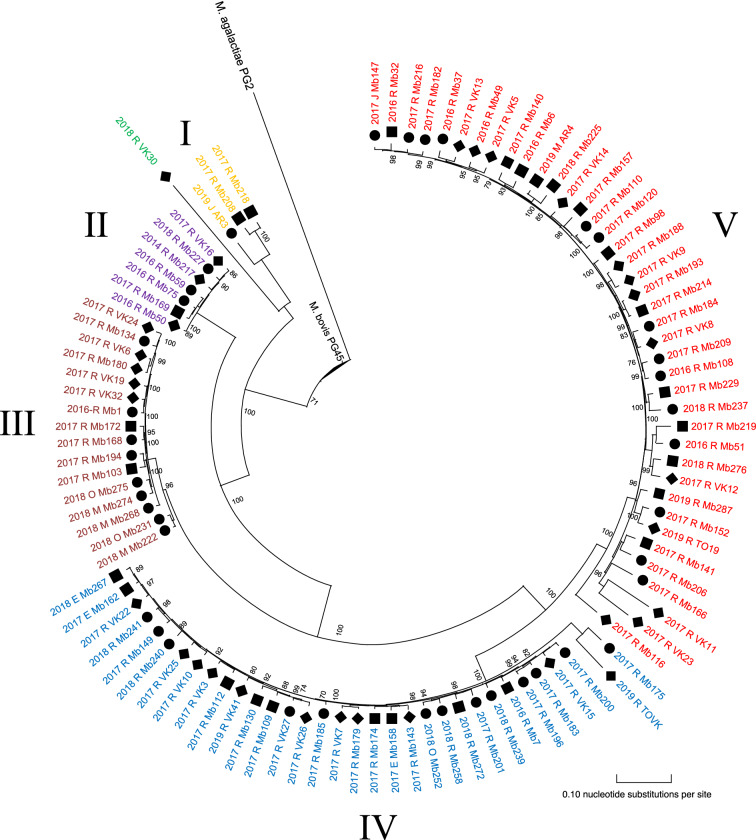
**SNP-based phylogenetic tree of 100** ***M. bovis*** **isolates from Belgian dairy, beef and veal farms.** The figure was created using MEGA-X software with *M. bovis* isolates obtained over 2014–2019. The tree was rerooted to *M. agalactiae* PG2, which was included as an outgroup. Clusters (I-V), and VK30 are represented by different colors. The designation of the isolates features the sector (■ dairy; ● beef; ♦ veal), year of isolation (2014–2019), affected organ (R: respiratory tract; M: milk; E: ear, J: joint and O: other) and sequence identification (see Additional file 1). The scale bar indicates the number of substitutions per site, and bootstrap values are represented on nodes.

Visual inspection of the phylogenetic analysis of 100 M*. bovis* isolates resulted in 5 clusters (I-V): 3 large clusters (n ≥ 10 isolates), 2 smaller clusters (n < 10), and 1 distinct strain (VK30) as shown in Figure 1. Cluster I to V contained 3, 7, 16, 33, and 40 M*. bovis* isolates, respectively. Inspection of pairwise SNP differences per cluster, showed more homogeny within clusters II and III (mean ΔSNPs of 87 and 316, respectively), compared with cluster I, IV and V (mean ΔSNPs of 834, 1027, and 1435) (Table 1). Mean SNP differences among within-cluster isolates and outlier calculations showed outliers within cluster III (Mb222, Mb231), IV (Mb201, Mb240, Mb175, TOVK) and V (Mb116, Mb166, VK11, VK23).

**Table 1:** **Pairwise SNP differences between** ***M. bovis*** **isolates within Belgian cluster I to V and VK30.**

Cluster	Min SNP	Max SNP	ΔSNP	Mean	SD
I	292	1126	834	843	427
VK30^a^	3221	3775	3436	3457	95
II	78	165	87	135	22
III	60	376	316	198	71
IV	76	1103	1027	245	251
V	77	1512	1435	445	267

Δ: difference between minimum (min) and maximum (max) pairwise SNPs; SD: standard deviation; ^a^VK30 was compared to cluster I to V.

**Figure 2:**
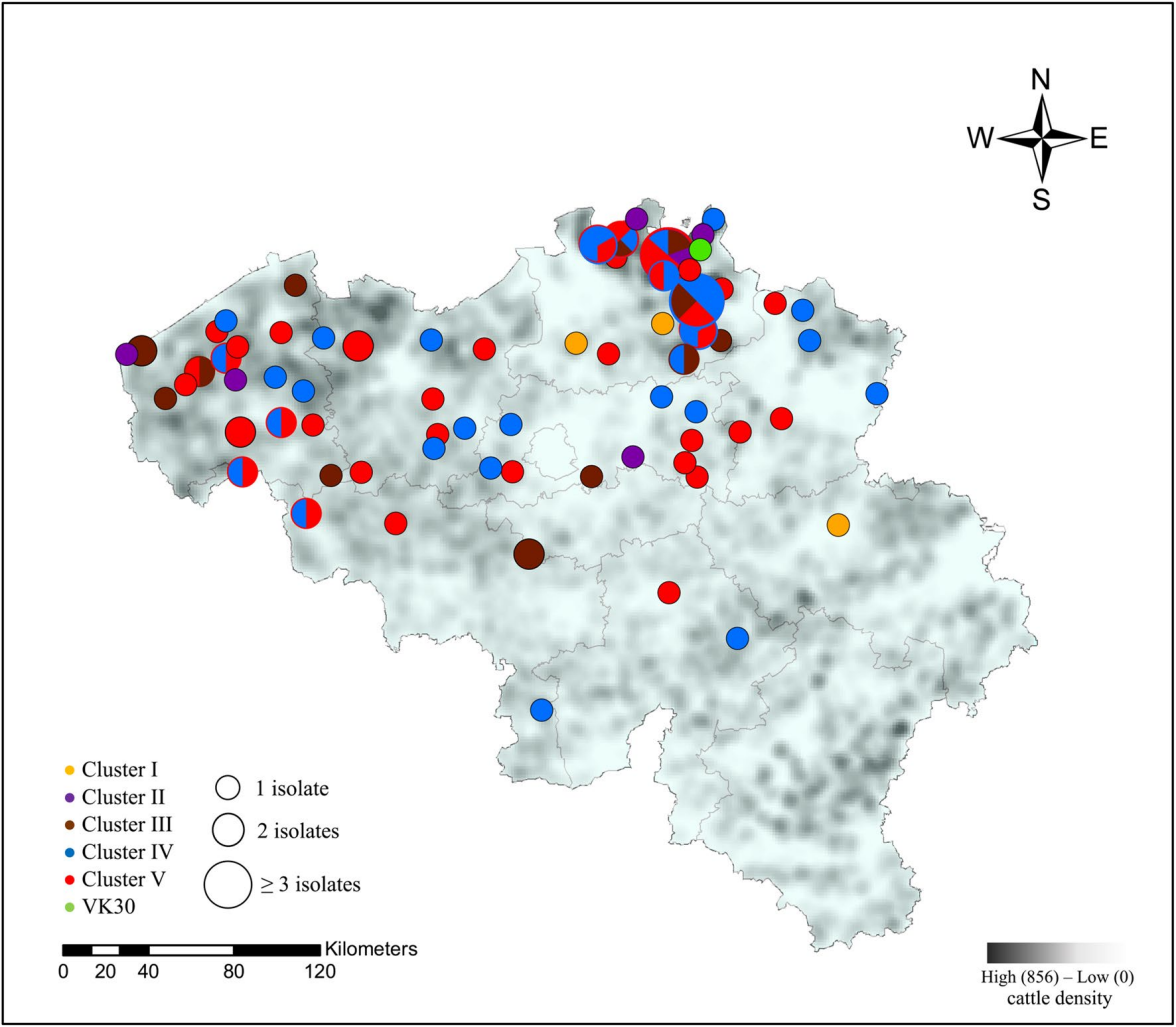
**Geographical distribution of different** ***M. bovis*** **clusters over 2014–2019 and cattle density in Belgium (2017).** The map was created using Esri®ArcMap™ (version 10.7.1) software. Clusters (I-V) are represented by different colors and the radius of the circle represents the number of isolates from one village. Mixed colors within one circle represent the presence of different clusters within one village.

Between and within clusters, no association could be observed for the different cattle sectors or year of isolation (Figure 1). Two different isolates from the same herd (veal) and same sampling period (Mb49 and Mb50) did not cluster together (II and V). All clusters persisted in Belgium for at least 1.5 consecutive years throughout the country. *M. bovis* strains isolated from the middle ear (n = 3) were clustered within cluster IV, while those obtained from milk, joint and other samples were scattered over different clusters (Figure 1). Finally, no clear association between geographic location of sampled farms and *M. bovis* clusters was observed, as shown in Figure 2.

### Phylogenetic analysis of *M. bovis* worldwide

**Figure 3:**
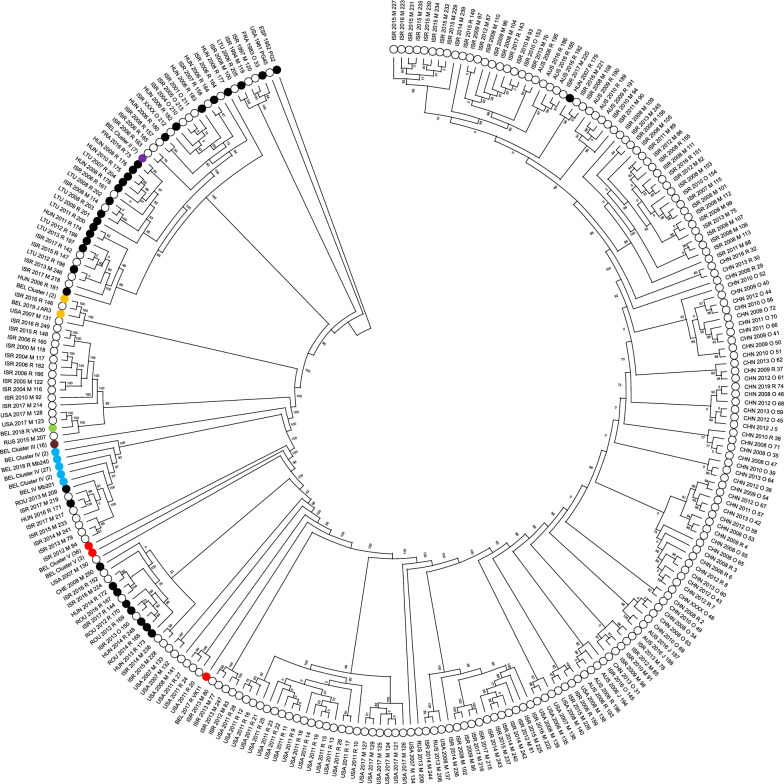
**SNP-based topology of 350** ***M. bovis*** **isolates in MEGA-X.** The tree was rerooted to *M. agalactiae* PG2 (EPS 1952 PG2), which was included as outgroup. Belgian clusters (I-V) were collapsed as far as possible and represented by different colors (I: yellow; II: purple; III: brown; IV: blue, V: red; VK30: green). The designation of the isolates contains the coded name of country of origin (ISO 3166-1; Alpha-3 code), continent of origin (● Europe; o Non-Europe), year of isolation (1952–2019), affected organ (R: respiratory tract; M: milk; E: ear, J: joint and O: other or unknown) and sequence identification (see Additional file 2) or the number of collapsed Belgian isolates between brackets. Bootstrap values are represented on nodes.

All 100 Belgian isolates were added to the worldwide phylogenetic tree. The percentage of the reference genome covered by all isolates, including the PG45 standard strain and the *M. agalactiae* outgroup strain PG2 was 39.3%, therefore 394 303 positions were found in all analyzed genomes. The minimum and maximum SNP differences among all *M. bovis* isolates including the reference strain, were 0 and 4871, respectively. Belgian clusters are situated in different parts of the phylogenetic tree worldwide (Figure 3). Cluster I is related to strains isolated in the USA (2007; mean ΔSNPs of 636) and Israel (2016; mean ΔSNPs of 1369). Cluster II is closely related to one recent French strain (2016; mean ΔSNPs of 171) and is situated in a larger cluster related to older strains from Israel and Eastern Europe (2001–2009; ΔSNPs < 200), and other more recently isolated strains from Israel and Eastern Europe (2011–2017; ΔSNPs < 500). Belgian cluster III and V do not cluster together with non-Belgian isolates, while cluster IV is closely related (ΔSNPs < 300 without cluster IV outliers) to *M. bovis* strains obtained in Israel (2012–2017) and Eastern Europe (2013–2016). VK11 remains an outlier that does not collate with the rest of cluster V. Consistent with Figure 1, VK30 is well separated from the other Belgian isolates and is very closely related (mean ΔSNPs of 171) to strains obtained from milk in the USA (2017).

## Discussion

In this study, one-hundred *M. bovis* isolates from different Belgian cattle sectors (beef, dairy or veal) were phylogenetically compared to investigate whether sector-specific strains exist and whether such strains are related to *M. bovis* strains previously isolated and sequenced worldwide.

In this study, we chose to apply the ONT long-read secquencing approach [36], because no default WGS approaches are defined for *Mycoplasma* spp. and short-read sequencing biases have been described for genomes with highly repetitive regions.

WGS approaches have become more attractive over the last years, as the cost for next-generation sequencing has significantly reduced. Single Nucleotide Polymorphism (SNP) analysis using Illumina short read data from *M. bovis* isolates already showed to be an effective way for *M. bovis* genotyping [24, 25]. Long-read nanopore sequencing (Oxford Nanopore Technologies) is known to create much faster results, and was recently applied in veterinary medicine as well [37, 38]. Yet, lower single read accuracies are currently obtained with ONT in comparison to Illumina [39]. Therefore, the implementation of long-read sequencing to generate *M. bovis* genome assemblies was verified, showing only on average 53 SNPs difference of the long-read approach with the publically available *M. bovis* PG45 genome, representing an acceptable error rate of 0.005%. As a result, the authors believe that nanopore sequencing is a highly accessible tool, allowing practical use outside academia in routine diagnostics and real time surveillance.

From the study results, several interesting observations were made. First of all, the obtained *M. bovis* isolates belonged to at least five different *M. bovis* clusters, of which three dominant clusters were identified. This is in agreement with an Israeli study based on WGS-SNP, where six clusters were observed of which three were dominant. Remarkably, one cluster contained more than 50% of the isolates in that study [25]. Several other studies also showed one or two dominant lineages, although different typing methods were used [13, 15, 18, 24, 40]. In contrast to Aebi et al. [41], where mostly herd-specific *M. bovis* isolates were seen in Switzerland, we observed close relatedness of *M. bovis* isolates over the different herds. This might be a result of more frequent purchasing cattle from different origins and transportation in Belgium, because 40% of cattle is transported at least once (and up to eight times) over a 5-year lifespan in Belgium [7, 16, 17, 26, 42]. As such, the higher heterogeneity observed in cluster I, IV and V compared with clusters II and III, may be caused by different rates of genetic drift between clonal lines [17].

Secondly, no sector-specific strains or clusters were identified in the present study, which does not entirely come as a surprise. In Belgium, veal calves are purchased from both dairy and beef farms, and fattened and slaughtered in specialized veal farms and slaughterhouses, respectively [27]. Also, approximately 15–20% of the farms in Belgium are mixed farms. Therefore, contact among different cattle sectors is intense. In addition, herd visitors and artificial insemination might play a role in spreading of *M. bovis* or introducing new strains on farms [43–45].

Thirdly, when we take a closer look at how the different clusters have spread over Belgium, no clear association with location was observed. This was also concluded in studies performed in the UK and USA [17, 23, 42]. Nevertheless, in the provinces of Antwerp and Western Flanders seem to be hotspots for *M. bovis* outbreaks. Besides a high number of local transports, Antwerp and the Flanders area are also the main gates for cattle import, which makes these areas predisposed for the introduction of new *M. bovis* strains [26]. Unfortunately, *M. bovis* genomes were not available for isolates obtained from the top import countries for Belgium, which are Germany and the Netherlands.

Although this study was not designed to draw definitive conclusions about year of isolation and affected organs, some preliminary observations can be made. For example, no association between *M. bovis* strain and year of isolation was observed. On the other hand, we saw that representatives of all clusters persisted for at least 1.5 consecutive years on Belgian territory. The persistence of strains within a country or herd has been described before [16, 24, 41]. Furthermore, shifts between dominant lineages from older to new strains have been reported before as well [13, 15, 46, 47]. Also, we did not observe an association between cluster and affected organ, which is in line with previous studies [14, 23, 24]. Yet, it was remarkable that all isolates obtained from the middle ear were clustered, which could suggest the middle ear as possible predilection site for certain *M. bovis* strains. However no definitive conclusions can be drawn as there were only few isolates obtained from this isolation site in the present study. In addition, we isolated different *M. bovis* strains (Mb49, Mb50) from two veal calves on the same farm at the same time. The observation of two different strains in one herd or even one animal has been described before [14, 16, 42, 48], in contrast to Arcangioli et al. [46], who isolated only one identical dominant profile in the same feedlot.

Finally, it was evident that Belgian isolates were mostly related to European and Israeli *M. bovis* isolates, even though only a few genomes of European *M. bovis* isolates have been published in the NCBI database. This seems plausible, as Belgian farmers mostly purchase cattle from European farms, while Israel also partly imports cattle from Eastern-Europe. The fact that Israeli isolates are often related to Chinese and Australian strains, is also due to import of cattle, as outlined in detail elsewhere [25, 40]. Some of the Belgian outlier strains were related to American isolates, which might be explained by the fact that *M. bovis* was first isolated in the USA and outbreaks in Europe were only seen years later. So, we can only speculate whether these outlier strains could have been imported or evolved geographically distinct from each other. Clusters of the Belgian isolates were not clustered exactly in the same way in the Belgian vs. the worldwide phylogenetic tree. A possible explanation could be the loss of overall coverage between the construction of both phylogenetic trees (51% for the Belgian to 40% worldwide). This might be due to (1) heterogeneity among isolates worldwide and/or (2) the use of genomes obtained by different laboratories, using different sequencing protocols, as the quality can be influenced by strain maintenance, DNA extraction, library preparation, sequencing, and the bioinformatics analysis [49].

In conclusion, multiple *M. bovis* clusters were circulating in Belgium in 2014–2019, and were persisting for several years. Neither the veal industry, nor any other cattle industry could be identified as source of strain persistence. Connections between dairy, beef and veal industry are intense and *M. bovis* appears to easily spread among these sectors. The *M. bovis* issues in the veal industry seem more likely the consequences of strain import from dairy and beef, rather than persistence of a limited number of veal specific strains. This information can contribute to better control and prevention *of M. bovis* infections by improved biosecurity.

## Supplementary information

**Additional file 1. Sequence identification and descriptives of Belgian** ***Mycoplasma bovis*** **isolates.**

**Additional file 2.** **Sequence identification and descriptives of 250 previously published** ***Mycoplasma bovis*** **sequences.**

## Data Availability

All *M. bovis* consensus genomes are available for download on the NCBI GenBank database under the BioProject PRJNA639688 and accession numbers (SAMN15246515-SAMN1524662). Sequencing summaries can be found in Additional file 1.

## Abbreviations

AFLP: amplification fragment length polymorphism
AMU: antimicrobial use
AP-PCR: arbitrarily primed PCR
MLST: multi-locus sequence typing
MLVA: multiple-locus variable-number tandem repeat
ONT: Oxford Nanopore Technologies
PFGE: pulsed-field gel electrophoresis
RAPD: random amplification of polymorphic DNA
SNPs: single Nucleotide Polymorphisms
WGS: whole genome sequencing
